# Prevalence and Molecular Characterization of *Cryptosporidium* in Goats across Four Provincial Level Areas in China

**DOI:** 10.1371/journal.pone.0111164

**Published:** 2014-10-24

**Authors:** Rongsheng Mi, Xiaojuan Wang, Yan Huang, Peng Zhou, Yuxuan Liu, Yongjun Chen, Jun Chen, Wei Zhu, Zhaoguo Chen

**Affiliations:** 1 Key Laboratory of Animal Parasitology of Ministry of Agriculture, Animal-borne Food Safety Research Center of Chinese Academy of Agricultural Sciences, Shanghai Veterinary Research Institute, Chinese Academy of Agricultural Sciences, Shanghai, China; 2 Jiangsu Co-innovation Center for Prevention and Control of Important Animal Infectious Diseases and Zoonoses, Yangzhou, China; 3 Lvxiang Town Agricultural Technology Extension Station of Jinshan District, Shanghai, China; 4 Tengzhou Animal Husbandry and Veterinary Technology Service Center, Tengzhou, China; Cornell University, United States of America

## Abstract

This study assessed the prevalence, species and subtypes of *Cryptosporidium* in goats from Guangdong Province, Hubei Province, Shandong Province, and Shanghai City of China. Six hundred and four fecal samples were collected from twelve goat farms, and the overall infection rate was 11.4% (69/604). Goats infected with *Cryptosporidium* were found in eleven farms across four provincial areas, and the infection rate ranged from 2.9% (1/35) to 25.0% (9/36). Three *Cryptosporidium* species were identified. *Cryptosporidium xiaoi* (45/69, 65.2%) was the dominant species, followed by *C. parvum* (14/69, 20.3%) and *C.* *ubiquitum* (10/69, 14.5%). The infection rate of *Cryptosporidium* spp. was varied with host age and goat kids were more susceptible to be infected than adult goats. Subtyping *C.* *parvum* and *C.* *ubiquitum* positive samples revealed *C. parvum* subtype IIdA19G1 and *C. ubiquitum* subtype XIIa were the most common subtypes. Other *C. parvum* subtypes were detected as well, such as IIaA14G2R1, IIaA15G1R1, IIaA15G2R1 and IIaA17G2R1. All of these subtypes have also been detected in humans, suggesting goats may be a potential source of zoonotic cryptosporidiosis. This was the first report of *C. parvum* subtypes IIaA14G2R1, IIaA15G1R1 and IIaA17G2R1 infecting in goats and the first molecular identification of *C.* *parvum* and its subtypes in Chinese goats.

## Introduction

*Cryptosporidium* is an intestinal protozoan parasite that reportedly infects humans and animals worldwide [1]. In neonatal ruminants, cryptosporidiosis is a leading cause of diarrhea and mortality, and causes farmers significant economic loss [2]. Numerous molecular biological techniques have detected *Cryptosporidium* species/genotype and subtypes and will improve our understanding of cryptosporidiosis transmission in man and animals [3].

The first *Cryptosporidium* infection in a goat was reported in Australia [4] and has since then been reported worldwide [2]. Few investigations report the molecular characterization of *Cryptosporidium* species/genotypes in goats. Belgium [5], Egypt [6], France [7], India [8], Italy [9], Norway [10], Spain [11], Sri Lanka [12], the Czech Republic [13], and Zambia [14] have all found *Cryptosporidium parvum* as the dominant species. Other species/genotypes, such as *C. hominis*, *C. xiaoi*, *C. ubiquitum*, *C. andersoni*, *Cryptosporidium* rat genotype and a novel *Cryptosporidium* genotype were also described in goats [10], [15]–[22].

Goats are economic resources in China. According to the National Bureau of Statistics, about 141.4 million goats were in China at the end of 2012 (http://data.stats.gov.cn/workspace/index?m=hgnd), but few studies exist identifying the molecular epidemiology of cryptosporidiosis in goats. In one study, three species (*C. ubiquitum*, *C. andersoni,* and *C. xiaoi*) were found in Henan Province and one species (*C. ubiquitum*) was found in Chongqing City [20]. In another report, *C. xiaoi* and a novel *Cryptosporidium* genotype were found in goats in Qinghai Province [17]. Given the sparse data on prevalence and molecular characterization of *Cryptosporidium* spp. in goats in China, the present study aimed to supplement the genetic characterization of *Cryptosporidium* spp. in partial provinces of China, to investigate the subtypes of *C. parvum* and *C. ubiquitum*, and to evaluate the potential threat of *Cryptosporidium* spp. in goats to human health.

## Materials and Methods

### Ethics Statement

The protocol of this work was approved by the Animal Care and Use of Chinese Academy of Agricultural Sciences, and authorized by the Animal Ethical Committee of Shanghai Veterinary Research Institute. All the fecal samples obtained from goat farms were permitted by the owners of farms.

### Sample Collection

Fresh fecal samples were collected from 12 goat farms across four provincial areas of China from November 2007 to September 2013. Two farms from Huizhou City (22°47′N, 114°27′E) are located in Guangdong Province (South China area), four from Gongan County (30°03′N, 112°13′E) in Hubei Province (Central China area), two from Tengzhou City (35°03′N, 117°08′E) in Shandong Province (East China area), and one from Chongming County (31°36′N, 121°40′E), one from Jinshan District (30°49′N, 121°36′E) and two from Fengxian District (30°55′N, 121°22′E) in Shanghai City (East China area). The weather across four provincial areas are different. Guangdong has a tropical and sub-tropical climate (warm and humid all year round), Hubei and Shanghai belongs to the sub-tropical monsoonal climates (hot, humid summers and generally mild winters), and Shandong has a warm-temperate monsoonal climate (hot, rainy summers and cold, dry winters) (http://en.wikipedia.org/wiki/Category:Geography_in_China_by_province). The flocks from all the farms were similar, and the goats were kept within fenced areas at daytime and in house with wooden, slatted floors or on the cement floor at night time. Most goats drank from water channels (source from water supply) and took native grasses. Six hundred and four samples were obtained directly from the rectums or immediately after the animals defecated using sterile gloves. The samples were sealed in bags, taken back to the laboratory and processed within a week. The goats involved in this study had not previously been examined for *Cryptosporidium* infection.

### DNA extraction, PCR amplification and sequence analysis

Genomic DNA was extracted from 300 mg of each faecal sample. All the samples were washed twice with sterile water and further purified using FastDNA SPIN Kit for Soil and the FastPrep Instrument (MP Biomedicals, Santa Ana, CA) in accordance with the manufacturer’s instructions. A two-step nested PCR of small subunit (SSU) rRNA gene was used to amplify the DNA fragment, as described by Xiao et al. [23], [24]. Positive and negative control samples were used for each PCR reaction. All secondary PCR products were sequenced as previously described [25]. Sequence analysis was guided using BLAST (http://blast.ncbi.nlm.nih.gov/Blast.cgi), and *Cryptosporidium* species/genotypes were identified preliminarily. A Neighbor-Joining (NJ) tree was constructed using/by MEGA 6 software (http://www.megasoftware.net/) to assess the genetic relationship between different *Cryptosporidium* species/genotype sequences [26]. The reference sequences of *Cryptosporidium* spp. were obtained from GenBank.

### Subtype identification

For *C. parvum*, a ∼400 bp fragment of the 60 kDa glycoprotein (gp60) gene was amplified by nested PCR as described by Sulaiman et al. [27]. For *C. ubiquitum*, a ∼950 bp fragment of the gp60 gene was amplified by nested PCR as reported by Li et al. [28]. The second PCR products were purified using AxyPrep DNA Gel Extraction Kit (Axygen Scientific, Hangzhou, China) and sequenced as SSU rRNA gene. The *Cryptosporidium* subtypes were identified as described by Sulaiman et al. [27] and Li et al. [28].

### Statistical analysis

*Cryptosporidium* spp. prevalence in different regions and ages was determined using IBM SPSS Statistics V21.0 for Windows (International Business Machines Corp, New York, USA). The differences were considered significant when *p*<0.05 by Pearson’s Chi-Square test (*χ* ^2^ test) analysis.

## Results

### *Cryptosporidium* infection in different farms

The overall prevalence of *Cryptosporidium* in four provincial level districts was 11.4% (69/604) (Table 1). The highest prevalence was found in Shandong Province (18/100, 18%), a very different result than in Guangdong Province (5/91, 5.5%) (*χ* ^2^ = 7.03, *p*<0.05). High prevalence could also be found in Hubei Province (13/111, 11.7%) and Shanghai City (33/302, 10.9%) (*χ* ^2^ = 3.52, *p*>0.05). There was also no significant difference between Guangdong Province, Hubei Province, and Shanghai City (*χ* ^2^ = 2.67, *p*>0.05).

**Table 1:** Prevalence and molecular characterization of *Cryptosporidium* spp. in different provincial areas.

			Species		
Province/City	No. Sample	No. Positive (%)	*C. xiaoi* (%)	*C. parvum* (%)	*C. ubiquitum* (%)
Guangdong	91	5(5.5)	3	0	2
Hubei	111	13(11.7)	10	3	0
Shandong	100	18(18.0)	18	0	0
Shanghai	302	33(10.9)	14	11	8
Total	604	69(11.4)	45(65.2)	14(20.3)	10(14.5)

Eleven goat farms tested positively for *Cryptosporidium*. The highest infection rate was in Gongan-4 farm (25.0%, 9/36) of Hubei Province, while the Gongan-3 farm had no *Cryptosporidium* positive samples (Table 2). Among the eleven farms with *Cryptosporidium* infection, the infection rates ranged from 2.9% to 25.0% (Table 2).

**Table 2:** Prevalence and molecular characterization of *Cryptosporidium* spp. in different farms.

				Species			Gp60 Subtypes	
Province/City	Farm	No. Sample	No. Positive (%)	*C. xiaoi* (%)	*C. parvum* (%)	*C. ubiquitum* (%)	*C. parvum*	*C. ubiquitum*
Guangdong	Huizhou-1	35	1(2.9)	1	0	0		
	Huizhou-2	56	4(7.1)	2	0	2		XIIa(2)
Hubei	Gongan-1	19	2(10.5)	0	2	0	IIaA14G2R1(1)Unknown(1)	
	Gongan-2	33	2(6.1)	2	0	0		
	Gongan-3	23	0	0	0	0		
	Gongan-4	36	9(25.0)	8	1	0	IIaA15G1R1(1)	
Shandong	Tengzhou-1	50	10(20.0)	10	0	0		
	Tengzhou-2	50	8(16.0)	8	0	0		
Shanghai	Chongming	155	18(11.6)	8	8	2	IIdA19G1(8)	XIIa(2)
	Fengxian-1	55	7(12.7)	1	0	6		XIIa(6)
	Fengxian-2	22	3(13.6)	3	0	0		
	Jinshan	70	5(7.1)	2	3	0	IIaA17G2R1(1)IIaA15G2R1(1)Unknown(1)	
Total		604	69(11.4)	45(65.2)	14(20.3)	10(14.5)		

### Prevalence of *Cryptosporidium* species

**Figure 1:**
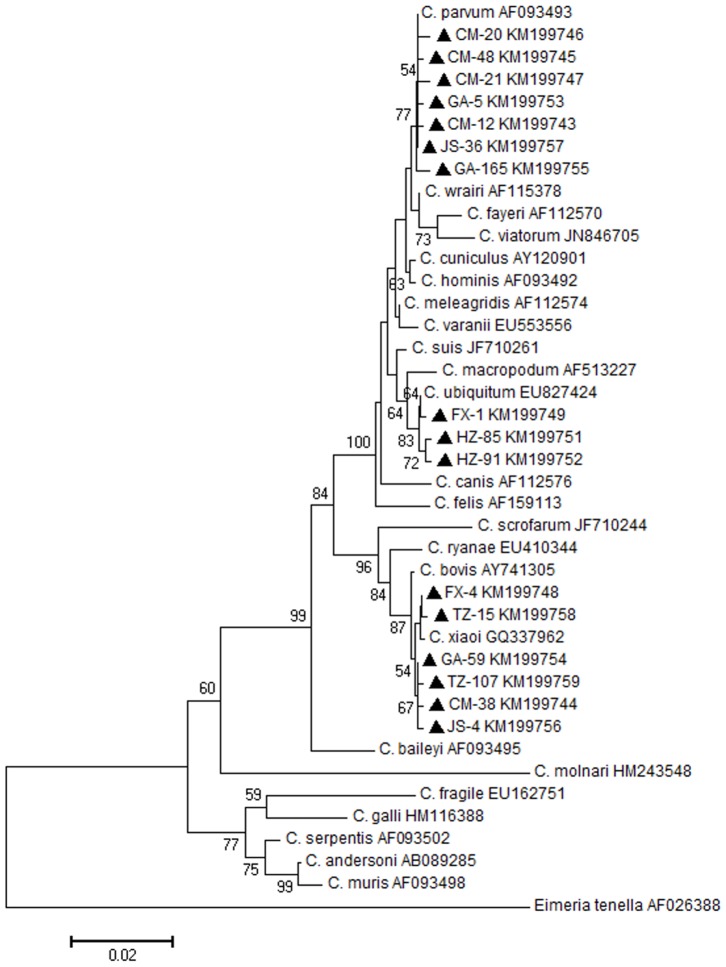
Phylogenetic tree of *Cryptosporidium* spp. constructed based on partial SSU rRNA nucleotide sequence. The phylogenetic tree was constructed using the neighbor-joining algorithm of the phylogeny program of MEGA 6.0. Bootstrap method via 1000 pseudo replicates was used to assess the reliability of the tree, and bootstrap value more than 50% were shown. ▴: Partial sequences obtained in the present study; CM: The sample from Chongming County; FX: The sample from Fengxian District; GA: The sample from Gongan County; HZ: The sample from Huizhou City; JS: The sample from Jinshan District; TZ: The sample from Tengzhou City.

All positive samples were sequenced and then searched using BLAST (http://blast.ncbi.nlm.nih.gov/Blast.cgi). Phylogenetic evolutionary analysis was determined using MEGA version 6 [26] (Fig. 1). Three *Cryptosporidium* species were detected, including *C. xiaoi* (45/69, 65.2%), *C. parvum* (14/69, 20.3%) and *C. ubiquitum* (10/69, 14.5%) (Table 2). They were found in 10 (Gongan-2, Gongan-4, Huizhou-1, Huizhou-2, Tengzhou-1, Tengzhou-2, Chongming, Fengxian-1, Fengxian-2 and Jinshan), 4 (Gongan-1, Gongan-4, Chongming and Jinshan), and 3 (Huizhou-2, Chongming and Fengxian-1) goat farms, respectively (Table 2). *C. xiaoi* was the most prevalent species in Gongan-4 (22.2%), while in Huizhou-2 and Chongming, *C. xiaoi* with *C. ubiquitum* and *C. xiaoi* with *C. parvum* were equally prevalent (Table 2). *C. ubiquitum* was the most prevalent species in Fengxian-1, and *C. parvum* was most prevalent in Gongan-1. Infections mixing two species were found in four farms (Gongan-4, Huizhou-2, Fengxian-1 and Jinshan), and infection from all three species was found in Chongming. The unique partial SSU rRNA gene sequences of *Cryptosporidium* spp. were submitted to the GenBank database under accession numbers KM199743 to KM199759.

### Prevalence of *Cryptosporidium* species in different goat age ranges

The infection rates of goats in four different age groups were investigated in the present study. The highest infection rate of *Cryptosporidium* spp. was observed in pre-weaned kids (11/55, 20.0%), followed by post weaned kids (34/227, 15%), yearling goats (23/306, 7.5%) and adult goats (1/16, 6.3%) (Table 3), with significant differences between each age groups (*χ* ^2^ = 11.9, *p*<0.05).

**Table 3:** Prevalence and molecular characterization of *Cryptosporidium* spp. in different host age groups.

			Species		
Age	No. Sample	No. Positive (%)	*C. xiaoi* (%)	*C. parvum* (%)	*C. ubiquitum* (%)
Pre-weaned kids	55	11(20.0)	6	5	0
Post weaned kids	227	34(15.0)	22	4	8
Yearling goats	306	23(7.5)	17	5	1
Adult goats	16	1(6.3)	0	0	1
Total	604	69(11.4)	45(65.2)	14(20.3)	10(14.5)

In addition to adult goats, *C. xiaoi* and *C. parvum* were found in three age groups. *C. ubiquitum* also found in three age groups except for pre-weaned kids. *C. xiaoi* (22/45, 48.9%) and *C. ubiquitum* (8/10, 80%) were mainly prevalence in post weaned kids, while *C. parvum* had a similar infection rates in three age groups (Table 3).

### Prevalence of *Cryptosporidium* subtypes

Twelve (85.7%) *C. parvum* positive samples were subtyped successfully based on the gp60 gene. Two subtypes, IIa and IId, were found. Four *C. parvum* IIa subtypes were found in 4 samples, including an IIaA14G2R1 from Gongan-1 farm and an IIaA15G1R1 from Gongan-4 farm of Hubei Province, and an IIaA15G2R1 and an IIaA17G2R1 from Jinshan farm of Shanghai City (Table 2). One IId subtype (IIdA19G1) was found in 8 samples from Chongming farm of Shanghai City (Table 2). All *C. ubiquitum* positive samples (10/10) were subtyped successfully, and all of them belonged to subtype XIIa (Table 2). The various sequences we acquired in this study were put in the GenBank database under accession numbers KM199736 to KM199742.

## Discussion

*Cryptosporidium* infection in goats has been reported all over the world in the past ten years, including in Belgium [5], Brazil [29], Cyprus [30], Egypt [6], England and Wales [31], France [15], [18], Greece [21], India [8], Italy [9], Malawi [32], Norway [10], Papua New Guinea [22], Spain [16], [33], [34], the Republic of Korea [19], Turkey [35], and Zambia [14]. However, most of these studies used microscopic examinations, immunofluorescence tests, or modified Ziehl-Neelsen staining techniques. Few reports used molecular methods, and the features of *Cryptosporidium* species/genotypes were still unclear.

In this study, the prevalence and molecular characterization of *Cryptosporidium* in goats across four provincial level areas in China were detected with nested PCR technique. Eleven goat farms had *Cryptosporidium* infection, and the infection rates ranged from 2.9% (1/35) to 25.0% (9/36). Only one farm (Gongan-3) from Hubei Province was not found *Cryptosporidium* infection. We found different provinces had different infection rates, and the highest prevalence was detected in Shandong Province (18/100, 18.0%), followed by Hubei Province (13/111, 11.7%), Shanghai City (33/302, 10.9%), and Guangdong Province (5/91, 5.5%). The total prevalence of *Cryptosporidium* spp. in goats from 12 goat farms was 11.4% (69/604), which was higher than the 4.4% (10/228) infection rate in Papua New Guinea used the same detection method [22], and lower than the 17.9% (980/5468) infection rate in goats summarized by Wang et al [20]. In China, partial provinces reported *Cryptosporidium* infection in goats, but the reported infection rates varied greatly. For instance, the infection rate was 35.7% (15/42) by immunofluorescence test (IFT) and 4.8% (2/42) by PCR approach in Wulan County of Qinghai Province [17]. The infection rates were 27.5% (14/51) by IFT and 15.7% (8/51) by PCR in Qinghai Province [36]. A recent survey reported the percentage of *Cryptosporidium* infection was 2.75% (28/1017) in Henan Province and 6.45% (16/248) in Chongqing City by microscopical detection [20]. The difference in infection rates between our survey and previous surveys may be affected by host age, examination methods, feeding levels and the raising density of animals.

All positive samples were sequenced successfully, and three species were identified in this study. *C. xiaoi* was the most common species (45/69, 65.2%), followed by *C. parvum* (14/69, 20.3%) and *C. ubiquitum* (10/69, 14.5%). The results were similar to a recent study in Greece, in which same *Cryptosporidium* species were observed in goat kids [21]. The *Cryptosporidium* species presented differently depending on geographical location. *C. xiaoi* by itself was found in Shandong Province, *C. xiaoi* and *C. parvum* mixed were found in Hubei Province, *C. xiaoi* and *C. ubiquitum* mixed were found in Guangdong Province. All three species were found in Shanghai City. These results agree with previous surveys in China, which detected *C. andersoni*, *C. ubiquitum* and *C. xiaoi* in Henan Province, *C. ubiquitum* in Chongqing City [20], and *C. xiaoi* and a new genotype in Qinghai Province [17], but we did not find *C. andersoni* in goats for this survey.

*C. xiaoi* (previously identified as *Cryptosporidium bovis-*like genotype) was first reported in a goat in Qinghai Province of China [17] and subsequently identified in countries like France, Norway, Papua New Guinea, and Spain [10], [15], [16], [22]. Recently, *C. xiaoi* was also found in Henan Province of China [20]. In this study, *C. xiaoi* as the dominant species was found in all the detected provincial areas and most of the detected goat farms. A previous French study found that *C. xiaoi* was the most common species in pre-weaned kids [15], and a recent study also reported a high prevalence of *C. xiaoi* infection in goat kids in Greece [21]. In China, *C. xiaoi* was also detected in sheep [37], [38] and a yearling yak [39]. A recent study reported that *C. xiaoi* was identified in two HIV/AIDS patients in Ethiopia [40], suggesting that it pose a potential threat to human health.

Most previous surveys found that *C. parvum* was the dominant species in goats [5], [8], [9], [11]. In this study, *C. parvum* was the second most prevalent species in goats. This agrees with a recent report from France in which *C. xiaoi* had a higher prevalence rate than *C. parvum* [15], and in Papua New Guinea where *C. hominis* was more prevalent than *C. parvum* [22]. To our knowledge, this was the first molecular identification of *C. parvum* infection in goats in China.

*C. ubiquitum* (formerly known as *Cryptosporidium* cervine genotype) as a zoonotic species has been reported in humans worldwide and in many animals, including ruminants, primates, and rodents, and has also been found in water [1], [27], [41], [42]. Few studies report it in goats. A recent survey in China have reported that *C. ubiquitum* was the predominant species in Chongqing City [20]. By contrast, we found *C. ubiquitum* was the least frequently detected species in goats. *C. ubiquitum* was found in only three goat farms (Huizhou-2, Chongming, and Fengxian-1). Interestingly, in Fengxian-1 farm of Shanghai City, *C. ubiquitum* was the dominant species.

Our survey, and previous surveys, revealed that young animals were more susceptible to infection than adult animals [29], [43]. Four age groups in goats were identified in this study, and the infection rate was decreased significantly with the increase of host age. All three species were found in post weaned kids and yearling goats, and *C. xiaoi* and *C. parvum* were found in pre-weaned kids, while *C. ubiquitum* as a unique species was identified in an adult goat. *C. xiaoi* was the major species, followed by *C. parvum* and *C. ubiquitum*, and these results were different than in previous studies. Most previous reports found *C. parvum* as the dominant species in goat kids [5.8,10,11,14]. Other studies in France and Spain listed *C. xiaoi* as the most common species in goat kids [15], [16], and *C. ubiquitum* as the unique species was found in peri-parturient goats in one flock in western France [18]. However, a recent study found that *C. parvum* and *C. xiaoi* existed in adult goats in Papua New Guinea [22], and *C. ubiquitum* and *C. xiaoi* were found in goats of all ages in China [20]. In this study, only one positive sample was detected in adult goats, it may be attributed to the small number of fecal samples. In future research, larger numbers of fresh samples of adult goats in these areas will be collected and detected to understand the relationship between the host age and *Cryptosporidium* species infection.

To date, only a few studies analyzed *C. parvum* subtypes in goats, and two subtype families (IIa and IId) were reported. The IIa family has been found in Belgium, Norway, and Papua New Guinea [5], [10], [22], and the IId family was only found in Europe (Belgium, Greece, Italy and Spain) [5], [9], [11], [21]. Similar to previous studies, the IIa and IId families were also found in China in this survey, and IId subtype family (8/12, 66.7%) was the dominant subtype in goats. This agrees with previous surveys that said goats were more susceptible to infection *C. parvum* IId subtypes in Belgium (8/11, 72.7%), Greece (2/2, 100%) and Spain (17/17, 100%) [5], [11], [21]. The distribution of *C. parvum* subtypes had geographic differences. IIa subtypes were found in the Gongan-1 (IIaA14G2R1) and Gongan-4 (IIaA15G1R1) goat farms of Hubei Province and in Jinshan farm (IIaA15G2R1 and IIaA17G2R1) of Shanghai City. The subtype IId was identified only in Chongming farm (IIdA19G1) of Shanghai City. The subtype IIdA19G1 had been detected in goats in previous studies from Spain [11] and in HIV-positive patients in China [44] and Portugal [45]. IIdA19G1 also had been found in calves in Henan Province [46], but had never been found in goats in China. Subtype IIaA15G2R1 was the most common subtype in calves and humans [3], [47]. It had been found in goats in Belgium [5] and Papua New Guinea [22]. In China, this subtype was also detected in lambs and yaks [38], [48] but not goats. Subtype IIaA14G2R1 was mainly found in calves in Europe, including in Belgium [49], England [50], Germany [51], and the Netherlands [52]. It was also found in humans in Ethiopia [40]. In China, this subtype had been found in yaks [48]. Other subtypes, IIaA15G1R1 and IIaA17G2R1, have been reported in humans in many previous studies. For example, subtype IIaA15G1R1 had been found in humans in Australia [53], Egypt [54], Kuwait [26], and Slovenia [55]. Subtype IIaA17G2R1 had been reported in Australia [53], [56], [57], Canada [58], Ethiopia [40], the United Kingdom [59], and the United States [60]. The three IIa subtypes detected in this study, including IIaA14G2R1, IIaA15G1R1 and IIaA17G2R1, are the first reported in goats. Taken together, all the *C. parvum* subtypes have been detected previously in humans, suggesting that goats may be involved in zoonotic transmission of cryptosporidiosis.

Recent research about *C. ubiquitum* subtyping that targeted the gp60 gene was reported by Li et al. [28], and 6 subtype families (XIIa-XIIf) were identified. In this study, all the *C. ubiquitum* positive samples were identified as XIIa, in accordance with a recent survey that asserted XIIa was the unique subtype in goats in Henan Province and Chongqing City [20]. This subtype was also found in a goat from Algeria [28]. A recent study found *C. ubiquitum* subtype XIIa not only existed in domestic and wild ruminants, but also commonly seen in humans [28], which suggests this subtype may be a potential source of *C. ubiquitum* infection between animals and humans.

In summary, the prevalence and molecular characterizations of *Cryptosporidium* species and subtypes in goats from 12 farms across four different provincial level regions of China indicate that *Cryptosporidium* spp. are common infections in goats in China. The overall infection rate was 11.4% and the highest infection rate was found in pre-weaned kids. *C. xiaoi*, five subtypes of *C. parvum,* and one subtype of *C. ubiquitum* were found in goats. To our knowledge, this was the first report of *C. parvum* subtypes IIaA14G2R1, IIaA15G1R1 and IIaA17G2R1 infecting in goats and the first molecular identification of *C. parvum* and its subtypes in Chinese goats. The zoonotic *C. parvum* subtype IIdA19G1 and *C. ubiquitum* subtype XIIa were the dominant subtypes in the present study, suggesting that monitoring goat populations for the presence of *Cryptosporidium* is important to public health.
